# Evaluation Design of New York City’s Regulations on Nutrition, Physical Activity, and Screen Time in Early Child Care Centers

**DOI:** 10.5888/pcd11.130431

**Published:** 2014-10-16

**Authors:** Andrew Breck, Ken Goodman, Lillian Dunn, Robert L. Stephens, Nicola Dawkins, Beth Dixon, Jan Jernigan, Jakub Kakietek, Catherine Lesesne, Laura Lessard, Cathy Nonas, Sarah Abood O’Dell, Thearis A. Osuji, Bernice Bronson, Ye Xu, Laura Kettel Khan

**Affiliations:** Author Affiliations: Andrew Breck, Bernice Bronson, National Foundation for the Centers for Disease Control and Prevention, Atlanta, Georgia; Ken Goodman, Robert L. Stephens, Nicola Dawkins, Jakub Kakietek, Catherine Lesesne, Sarah Abood O’Dell, Thearis A. Osuji, Ye Xu, ICF International, Atlanta, Georgia; Lillian Dunn, Cathy Nonas, New York City Department of Health and Mental Hygiene, New York, New York; Beth Dixon, New York University, New York, New York; Jan Jernigan, Laura Kettel Khan, Centers for Disease Control and Prevention, Atlanta, Georgia; Laura Lessard, Arcadia University, Glenside, Pennsylvania.

## Abstract

This article describes the multi-method cross-sectional design used to evaluate New York City Department of Health and Mental Hygiene’s regulations of nutrition, physical activity, and screen time for children aged 3 years or older in licensed group child care centers. The Center Evaluation Component collected data from a stratified random sample of 176 licensed group child care centers in New York City. Compliance with the regulations was measured through a review of center records, a facility inventory, and interviews of center directors, lead teachers, and food service staff. The Classroom Evaluation Component included an observational and biometric study of a sample of approximately 1,400 children aged 3 or 4 years attending 110 child care centers and was designed to complement the center component at the classroom and child level. The study methodology detailed in this paper may aid researchers in designing policy evaluation studies that can inform other jurisdictions considering similar policies.

## Introduction

In March 2006, the New York City Department of Health and Mental Hygiene (DOHMH) published a report on the prevalence of obesity among young children who attended Head Start programs in New York City (1). In response, the New York City Board of Health adopted revisions to Article 47 of the New York City Health Code governing licensed group child care centers in an effort to reduce early childhood obesity (2) (Box). Beginning January 1, 2007, centers were required to comply with the new regulations that restrict provided beverages, set minimum amounts for physical activity, and limit television viewing. We describe the multi-method approach developed and implemented to evaluate New York City’s regulations of beverages served, physical activity, and screen viewing in group child care centers. This article provides a detailed description of the evaluation design and methods. The results of this evaluation are presented in the multiple manuscripts included in this special collection (3–6).

**Box. Child Care Regulations in Article 47 of the New York City Health Code**

- Children cannot be served beverages with added sweeteners.
- Children can only be served juice that is 100% juice, and no more than 6 oz per day and only served to children 8 months old or older.
- Children 2 years old or older can only be served milk that is 1% milk fat or less.
- Children must have water available and easily accessible throughout the day.
- Children 1 to 3 years old must have at least 60 minutes of physical activity every day.
- Children 3 years old or older must have at least 60 minutes of physical activity every day, 30 minutes of it guided and structured.
- Children younger than 2 years old are not permitted to watch television.
- Children 2 years old or older are allowed a maximum of 60 minutes of television viewing per day (restricted to educational programs or those that engage child movement).

The multi-method approach to evaluate New York City’s licensed group child care centers regulations involved 2 components. The first evaluation component (Center Evaluation Component) was designed to evaluate whether compliance was associated with center and staff characteristics. The second evaluation component (Classroom Evaluation Component) was designed to evaluate whether compliance was associated with staff and children’s behavior. Institutional review boards of DOHMH and ICF International reviewed and approved all components of the evaluation. We present 1) the sampling methods and sample characteristics for the center and the classroom components of the study, 2) the instruments and data collection methods for the center component, and 3) the instruments and data collection methods for the classroom component.

## Sample Selection

The sampling universe included all 1,656 DOHMH-licensed group child care facilities. Three District Public Health Offices (DPHOs) include catchment areas (ie, geographic areas within New York City that have high risk factor and disease burden and therefore increased need for public health services) that consist of low-income, high-risk neighborhoods, specifically East and Central Harlem, the South Bronx, and East and Central Brooklyn. It was thought that centers in these low-income neighborhoods might have the greatest challenge in complying with the new regulations, and they are eligible for training and technical assistance from the DPHO at no cost. To account for the differences in technical assistance received, the sample was stratified according to DPHO status (ie, whether centers were in a DPHO neighborhood or not).

Of the 1,656 licensed group child care facilities in New York City, 311 were in DPHO neighborhoods. Although 97% (301 out of 311) of the DPHO centers were in census tracts with 40% or more of families with incomes at 200% of the federal poverty threshold or below, only about 41% (549 out of 1,345) of the non-DPHO centers were in neighborhoods with such high poverty levels. To ensure comparability of DPHO and non-DPHO centers, only centers from high-poverty areas were randomly included in the sampling frame (300 in DPHO and 350 in non-DPHO catchment areas). The final center component sample consisted of a random sample of 130 centers in DPHO and 130 centers in non-DPHO catchment areas (Figure).

**Figure:**
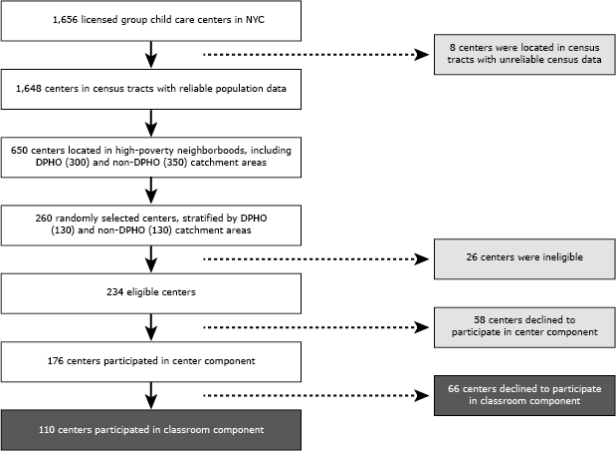
Selection of New York City (NYC) licensed group child care centers for Center Evaluation Component (fall 2009) and Classroom Evaluation Component (spring 2010) selected through a stratified sampling approach. Abbreviation: DPHO, District Public Health Office.

Center eligibility was based on 4 criteria: 1) had a classroom of at least ten 3- to 4-year-old children, 2) had at least 2 teachers of 3- to 4-year-old classrooms, 3) did not exclusively serve children with special needs, and 4) was not closing or had not already closed. Twenty-six centers were excluded because they did not meet these criteria, and an additional 58 centers elected not to participate.

The center component was completed in 176 child care centers (93 in DPHO and 83 in non-DPHO catchment areas). Most centers were in the Bronx, Brooklyn, or Manhattan, supporting the DOHMH’s interest in examining how the additional training and technical assistance provided affected a center’s capacity to comply with the regulations (Table 1). The 176 group child care centers that participated in the center component were all invited to participate in the classroom component; 110 centers agreed to participate (65 in DPHO and 45 in non-DPHO catchment areas). Each center for the center component received a $100 gift card to an educational resource retailer. Each of the classroom component centers received another $100 gift card, and classroom teachers received a $25 American Express gift card.

**Table 1:** New York City Child Care Center Sample Characteristics, Fall 2009 and Spring 2010

Sample Characteristics	Center Component, n = 176	Classroom Component, n = 110
**Borough, n (%)**		
Bronx	43 (24.4)	35 (31.8)
Brooklyn	75 (42.6)	40 (36.4)
Manhattan	44 (25.0)	28 (25.5)
Queens	13 (7.4)	7 (6.4)
Staten Island	1 (0.6)	0
**Capacity, n (%)**		
Head Start^a^	48 (27.2)	34 (30.9)
CACFP^b^	144 (81.8)	97 (88.2)
Center part of a larger agency (not part of ACS^c^ or Head Start)	69 (39.2)	50 (45.4)
**Director’s tenure, n (%)**		
Less than 3 years	46 (26.1)	28 (25.5)
3–5 years	23 (13.1)	17 (15.4)
More than 5 years	107 (60.8)	65 (59.1)
**Director’s educational attainment, n (%)**		
No bachelor’s degree	8 (4.5)	5 (4.5)
Bachelor’s degree	19 (10.8)	12 (10.9)
Graduate or professional degree	149 (84.7)	93 (84.6)
Indoor physical activity facilities	61 (34.7)	38 (34.5)
**Outdoor physical activity facilities, n (%)**		
Private	127 (72.2)	79 (71.9)
Shared	32 (18.2)	21 (19.1)
**Training and technical assistance, n (%)**		
Located in a DPHO^d^ catchment area	93 (52.8)	65 (59.1)
SPARK!^e^ participant	153 (86.9)	102 (92.7)
EWPH^f^ participant	54 (30.7)	44 (40.0)
TOT^g^ participant	19 (10.8)	15 (13.6)
Director trained for SPARK!	93 (52.8)	66 (60.0)
**Center characteristics, mean (standard deviation)**		
Average classroom size (3- to 4-year-olds)	16.1 (3.9)	16.5 (3.5)
No. of hours centers were open	10.0 (1.2)	10.0 (1.0)
Food service staff per center	2.0 (1.4)	2.3 (1.3)
Student-teacher ratio	5.7 (3.0)	5.8 (3.0)
Teaching staff that terminated employment compared with total teaching staff (eg, turnover ratio per center, 2008–2009)	0.13 (0.2)	0.09 (0.15)
**Training and technical assistance, mean (standard deviation)**		
No. of physical activity–related training programs other than SPARK! and EWPH	0.3 (0.5)	0.24 (0.47)
No. of nutrition-related training programs other than SPARK! and EWPH	0.7 (0.6)	0.78 (0.63)
Teachers trained in first SPARK! workshop	8.7 (9.1)	10.1 (9.8)
Teachers trained in second SPARK! workshop	1.2 (3.6)	1.1 (3.98)
Teachers trained in TOTs	0.5 (2.4)	0.5 (2.4)

Abbreviations: CACFP, Child and Adult Care Feeding Program; ACS, Administration for Children’s Services; DPHO, District Public Health Office; SPARK!, Sports, Play, and Recreation for Kids!; EWPH, Eat Well Play Hard; TOT, Training of Teachers; DOHMH, New York City Department of Health and Mental Hygiene.

a Head Start is a comprehensive developmental program of the Administration for Children and Families within the US Department for Health and Human Services for preschool-aged children and their families whose household income is below the federal income poverty threshold.

b CACFP is a program of the US Department of Agriculture that administers federal grants to state health departments to provide nutritious meals and snacks to low-income individuals.

c ACS is New York City government’s child welfare agency.

d DPHOs are a program of the DOHMH that target resources to high-need neighborhoods in the South Bronx, East and Central Harlem, and East and Central Brooklyn.

e SPARK! is a physical activity training program that DOHMH provides free of charge to licensed group child care centers.

f EWPH is a childhood obesity initiative of the New York State Department of Health. EWPH intervention involves a 6-week training program provided free of charge by the DOHMH to child care centers where at least 50% of the enrolled students are eligible for free or reduced-price meals.

g TOT is a DOHMH technical assistance program that provides child care center staff the skills necessary to lead the EWPH nutrition and physical activity curriculum in their classrooms.

Although the focus of the study was 3- to 4-year-old children (94% of final sample), because the age of children within a classroom varied, our analysis included children 2 to 6 years of age (n = 1,427). Validated accelerometer cut points only exist for children older than 3 years old, therefore we excluded children less than 2 years 10 months of age (n = 29 aged 34–36 months). We also excluded children who were more than 6 years of age (n = 1). Most (92%) of the children were Hispanic or non-Hispanic black (Table 2).

**Table 2:** New York City Child Care Child Participant Characteristics, Spring 2010 Classroom Component (N = 1,427)

Characteristic	n (%)
**Age, y**	
2	32 (2.2)
3	871 (61.0)
4	467 (32.7)
5	57 (4.0)
**Race/ethnicity**	
Hispanic or Latino	632 (44.3)
White	14 (1.0)
Black or African American	669 (46.9)
American Indian	2 (0.1)
Asian	34 (2.4)
Not Hispanic or Latino (other race)	63 (4.4)
Missing	13 (0.9)
**Sex**	
Female	754 (52.8)
Male	672 (47.1)
Missing	1 (0.1)
**Weight status** a	
Underweight	68 (4.8)
Healthy weight	924 (64.8)
Overweight	214 (15.0)
Obese	210 (14.7)
Missing	11 (0.8)

a Weight status categories are determined by body mass index (BMI, kg/m^2^) *z* score. Underweight: BMI <5th percentile; healthy weight: BMI 5th percentile to <85th percentile; overweight: BMI 85th percentile to <95th percentile; obese: BMI ≥95th percentile.

## Center Evaluation Component — Staff Interviews and Site Inventory

The center component of the evaluation involved interviewing staff and conducting a site inventory. Center-level compliance with the regulations was assessed through a review of center records, the site inventory, and in-person interviews with center directors, teachers, and food service staff. DOHMH staff and sanitarians were interviewed on training, technical assistance, and enforcement of the regulations. Standardized, written data collection protocols were developed. In addition, DOHMH provided center-level data regarding dates of participation in DOHMH-provided physical activity and nutrition training programs, such as Sports, Play, and Recreation for Kids! (SPARK!), Eat Well Play Hard (EWPH), and EWPH Training of Teachers. These training programs are described in detail in Nonas et al (3) in this issue.

### Instruments

The center component interview instruments were designed to assess center level compliance and the degree to which center staff members were familiar with the regulations. In addition, the instruments were designed to identify barriers to compliance with the regulations and the effect that DOHMH training had on staff awareness of the regulations. Teachers selected for participation were identified by center directors. Interview instruments were designed for each type of staff position and adapted from existing instruments (7,8). The site inventory recorded the types of beverages present in food storage and preparation areas, location and number of televisions, the availability of indoor and outdoor play space, and characteristics of the center’s neighborhood that could facilitate physical activity, such as access to safe places to play (eg, a neighborhood park). Table 3 lists background resources and evaluation instruments developed for each evaluation component. The center component instruments were pilot tested by project team members at 2 child care centers and revised in advance of data collector training. The results of these pilot tests are not included in analyses.

**Table 3:** Overview of New York City Child Care Evaluation Data Collection Instruments and Resources

Instrument/Data Source	Purpose/Content/Strengths and Weakness of Data Source	Unit of Analysis
**Center Evaluation Component**		
DOHMH administrative data	Primarily used for sampling stratification (center address and location relative to DPHO catchment areas), and indicators of center participation in DOHMH training programs. Low data collection cost and burden.	Center
DOHMH staff interviews	Provide context for the setting in which the beverage and physical activity regulations are implemented by interviewing staff responsible for providing child care center training and/or enforcing regulations. Low data collection cost and burden.	Context
Director, teacher and food service staff interviews	Respondents’ knowledge of and reported compliance with New York City’s regulations for beverages, physical activity, and screen time at child care centers. Moderate data collection cost and burden on center staff and time required.	Center
Site inventory	Beverages present in the child care center food storage and preparation areas and the centers’ physical activity environment. Low data collection cost and burden.	Center
**Classroom Evaluation Component**		
Child information form	Director reported child birth dates, sex, race/ethnicity, start date in the center, and number of days per week and number of hours per day attending the day care. Moderate data collection cost and burden on center staff and time required.	Center
General observation form	Episodes, context, and environment of physical activity in selected classroom. High data collection cost and time required.	Classroom
Child accelerometry form	Height and weight and accelerometer start and stop times for children in selected classroom. High data collection cost and time required.	Child
Nutrition observation form	Components of every meal and snack provided to children in selected classroom. High data collection cost and time required.	Classroom
Mealtime observation form	Quantity of meal and snack items served to and consumed by observed children in selected classroom. High data collection cost and time required.	Child

Abbreviations: DOHMH, New York City Department of Health and Mental Hygiene; DPHO, District Public Health Office.

### Data collection

For the center component, 10 data collectors were trained in October 2009. This training focused on the purpose and methods of the evaluation, interview surveys, use of the SPSS Data Collection Interviewer Desktop, Version 5.50.000.5009 (IBM, Inc), and a supervised site visit. The center component data collection occurred from October 2009 to January 2010.

In January and February 2010, ten in-depth semistructured telephone interviews to provide context were completed with DOHMH staff members who enforce regulations and oversee various training programs but were not part of the evaluation team. Results of the center component are reported by Lessard et al (4) and Kakietek et al (5) in this issue.

## Classroom Evaluation Component — Classroom and Child Direct Observation

The classroom component was an observational and biometric study of a subsample of the centers participating in the center component to determine whether compliance was associated with staff and child behavior. Classroom-level compliance with the regulations and child behavior was assessed through observation of beverages served, access to water throughout the day, and physical activity opportunities offered as well as documentation of center characteristics that support physical activity opportunities. Child-level outcomes were examined by using observed beverages consumed during meals and snacks and intensity and duration of physical activity measured by accelerometry. The classroom serving children aged 3 to 4 years was selected for the Classroom Evaluation Component. If a center had more than 1 classroom serving children aged 3 to 4 years, one of these classrooms was randomly selected for participation in the study.

### Instruments and measurement

The classroom component included 2 days of classroom-level and child-level observation of foods and beverages provided and physical activity opportunities offered to children between 8 AM and 5 PM. Child-level measures included amounts of foods and beverages served and consumed, child’s height in centimeters and body weight in kilograms, and amount and intensity of physical activity each child achieved via accelerometry. Demographic information was collected by using a Child Information Form; data collected included date of birth, sex, race/ethnicity, and start date at the center.

A General Observation Form was created to record the episodes and context of physical activity, staff behavior related to foods provided and physical activity, children’s access to water, and classroom staff participation in training. This instrument was adapted from forms used by DOHMH to verify compliance by their sanitarians and from other validated instruments (9–12). An Accelerometry Form was used to record the time the accelerometer (Actigraph GT3X) was placed on and removed from the child, and the child’s height and body weight were recorded on the General Observation Form (9,13,14). The Nutrition Observation Form was used to record food and beverage components of every meal and snack, including service style and preparation. This instrument was used to record staff behavior, such as providing second servings without the child asking, encouraging the child to try new foods, and drinking or eating less healthful foods in front of the children. The Mealtime Observation Form was used to collect information on all food and beverages served to and consumed by the children. The dietary observation instruments were adapted from existing literature on dietary assessment (12,15–17).

The classroom component instruments and protocols were pilot-tested at 1 randomly selected, eligible group child care center and revised. The results of these pilot tests were not included in the evaluation results.

### Data collection

Twenty data collectors were trained over a 5-day period that included classroom-based training and site visits. Two-day site visits were conducted between April and June 2010.

On arrival at the centers, data collectors measured height and weight for each child who had parental consent to participate. Height was measured in centimeters by using a portable stadiometer (Seca 213) and weight was measured in pounds by using a portable scale (Seca Clara 803). Each child was measured twice, and the data were recorded to ensure accuracy on the first day of data collection. Anthropometric measurements were averaged and a SAS program (SAS Institute, Inc) developed by the Centers for Disease Control and Prevention (CDC) was used to calculate BMI *z* scores. Children wore the GT3X Actigraph accelerometers for the duration of the 2-day observation period. Some children had 1 day of accelerometry data because they either refused to wear the accelerometer on the first or second day or did not attend the child care center on the second day of data collection.

Using the nutrition observation form, data collectors recorded all food served in the classroom over the 2 days. During a typical day, meals often included breakfast or morning snack, lunch, and afternoon snack. Three unique children were randomly selected per day. These children did not have to have parental consent to be observed. This resulted in a total of 6 unique child dietary records per center. Results of the classroom component are reported in Kakietek et al (5) and Stephens et al (6) in this issue.

## Discussion

This evaluation is the first to measure compliance with beverage and physical activity regulations in a large sample of New York City child care centers in low-income neighborhoods. The data collected using this multi-method approach resulted in the creation of compliance scores for each center for the center and the classroom components, the calculation of a consistency of compliance score based on data from both the center and classroom components, and an analysis of the factors that are associated with compliance. By using mixed methods, we triangulated center-level and child-level data sources and conducted a multi-level assessment of the association between consistency of implementation of the New York City regulations and child behavior.

### Limitations

A limitation of this evaluation is the absence of pre-intervention center-level and child-level data. The use of a post cross-sectional evaluation design limits our ability to assess whether the adoption of the new regulations spurred child care centers in New York City to improve their policies and practices regarding beverages, physical activity, and screen time. Also, even though the center component and the classroom component were conducted within a close time period, they were not conducted simultaneously, so it cannot be assumed that the regulation was being implemented the same way. Another potential limitation is that compliance measures were partially based on self-reported data (eg, the staff interviews), which are subject to recall bias and social desirability in responses. In addition, it is possible that the teachers surveyed in the center component were not the same teachers observed or interviewed in the classroom component, leading to a potentially large intra-center variation.

It is also possible that the study sample was biased because of nonrandom refusal to participate. Although centers included in the center component sample were selected randomly from a sampling frame, about one-quarter of the eligible sampled centers (58 out of 234) refused to participate. Similarly, 66 centers that participated in the center component of the study opted to not participate in the classroom component. When compared with centers that participated in the center component only, centers that participated in the classroom component reported significantly more of the following: participation in CACFP, being a part of a larger parent agency, having a dedicated food service staff, being in DPHO areas, and participating in DOHMH training programs such as SPARK! and EWPH. It was possible that centers with poor compliance with the regulations were less likely to participate than centers with better compliance.

### Strengths

Despite the limitations noted above, this study has numerous unique strengths. At the time of this evaluation, New York City was one of the only major municipalities to have strong regulations for beverages, physical activity, and screen time at licensed group child care centers. As a result, this study design was constructed specifically for the New York City regulatory and training environment. DOHMH staff members with intimate knowledge of the history and intensity of training and technical assistance provided to the centers before and after implementation of the regulations were involved in the study design. Additionally, the study sample focused on low-income neighborhoods that could have more difficulty than high-income neighborhoods in implementing the regulations.

### Conclusions

The unique design of this evaluation contributes to the field both through findings and evaluation of practice. Although the results of this evaluation are limited to New York City’s metropolitan, urban, low-income communities, these results have potential importance for communities across the nation. The original purpose of the 2 data collection methods for assessing regulation compliance was not to compare or contrast the results for assessing compliance, but the resulting data identified important differences in measuring levels of compliance when using different methods. Practitioners and researchers alike can benefit from understanding the differences (4). Furthermore, the use of accelerometry in the assessment of physical activity fills a gap in the field’s knowledge of intensity of activity among children younger than 6 years old, for which there are no federal recommendations. Additional analyses of these data have the potential to add to the knowledge of the type and intensity of children’s physical activities in structured and unstructured play.

Our use of multiple data collection methods to examine regulatory compliance in group child care environments contributes to the evaluation field because there are few studies systematically examining compliance and there is increasing demand for methods to assess policy implementation. This evaluation not only documented the extent of compliance by using a variety of methods but also identified factors that may affect a center’s ability to comply. To further contribute to the field, future research might examine topics such as inter-rater reliability of observations of environment and child behavior and, in particular, the validity of using self-report data compared with direct observation data on compliance with nutrition and physical activity regulations. Finally, we hope the methods outlined here will provide guidance for future evaluations that build on this work.
